# PAL – parallel active learning for machine-learned potentials†

**DOI:** 10.1039/d5dd00073d

**Published:** 2025-06-22

**Authors:** Chen Zhou, Marlen Neubert, Yuri Koide, Yumeng Zhang, Van-Quan Vuong, Tobias Schlöder, Stefanie Dehnen, Pascal Friederich

**Affiliations:** a Institute of Theoretical Informatics, Karlsruhe Institute of Technology Kaiserstr. 12 76131 Karlsruhe Germany pascal.friederich@kit.edu; b Institute of Nanotechnology, Karlsruhe Institute of Technology Kaiserstr. 12 76131 Karlsruhe Germany; c Institute for Physical Chemistry, Karlsruhe Institute of Technology Kaiserstr. 12 76131 Karlsruhe Germany

## Abstract

Constructing datasets representative of the target domain is essential for training effective machine learning models. Active learning (AL) is a promising method that iteratively extends training data to enhance model performance while minimizing data acquisition costs. However, current AL workflows often require human intervention and lack parallelism, leading to inefficiencies and underutilization of modern computational resources. In this work, we introduce PAL, an automated, modular, and parallel active learning library that integrates AL tasks and manages their execution and communication on shared- and distributed-memory systems using the Message Passing Interface (MPI). PAL provides users with the flexibility to design and customize all components of their active learning scenarios, including machine learning models with uncertainty estimation, oracles for ground truth labeling, and strategies for exploring the target space. We demonstrate that PAL significantly reduces computational overhead and improves scalability, achieving substantial speed-ups through asynchronous parallelization on CPU and GPU hardware. Applications of PAL to several real-world scenarios – including ground-state reactions in biomolecular systems, excited-state dynamics of molecules, simulations of inorganic clusters, and thermo-fluid dynamics – illustrate its effectiveness in accelerating the development of machine learning models. Our results show that PAL enables efficient utilization of high-performance computing resources in active learning workflows, fostering advancements in scientific research and engineering applications.

## Abstract 2

An automated, modular, and parallel active learning (PAL) library that integrates AL tasks and manages their execution and communication on shared- and distributed-memory systems using the Message Passing Interface (MPI).

## Graphical abstract

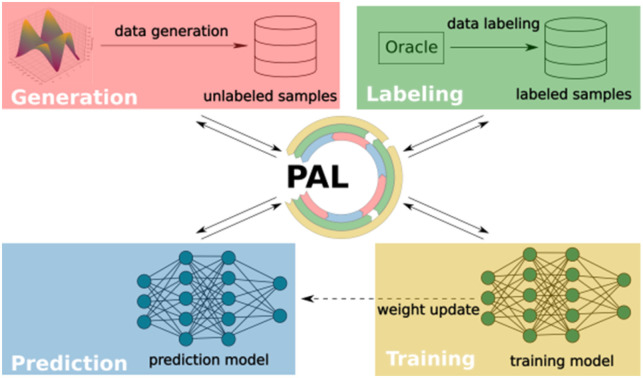

## 1 Introduction

In many ML applications, the primary goal of data generation is to create a diverse dataset that comprehensively represents the relevant data distribution, ensuring stability and reliability during deployment.^1^ In contrast, scientific applications often require ML models to operate in exploratory settings, where discovering novel and unseen inputs is expected or even desired.^2–4^ ML models trained on limited initial datasets often struggle to generalize effectively to newly encountered instances outside the training distribution. Continuously updating the model by labeling portions of the explored input space and retraining is inefficient, as it frequently adds redundant data points to the training set without significantly improving generalization accuracy. To address this challenge, active learning (AL) has been introduced as an efficient approach to building accurate and reliable models while minimizing queries to an oracle – a computational method or experimental process capable of providing ground truth labels.^5^ By iteratively selecting the most informative instances for labeling, AL reduces the overall cost of training set generation and enhances model performance.^6–8^

**Fig. 1:**
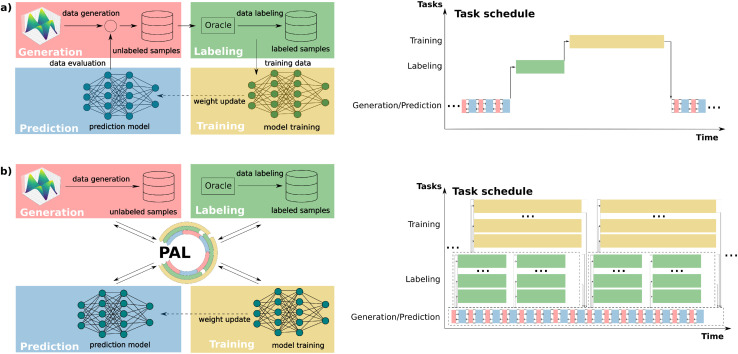
Comparison of (a) a conventional (serial) active learning and (b) our parallel active learning workflow PAL. (a) Classical active learning workflow, in which different tasks, *i.e.* exploration of the input space using generation and prediction kernels, labeling of the samples using the oracle kernel, and training of the ML model, are performed iteratively and sequentially. (b) PAL modularizes, decouples, and parallelizes data generation, oracle labeling, and ML training processes.

One particularly important application of active learning is developing new machine-learned potentials, which are often trained on quantum mechanical calculations to map three-dimensional atomic coordinates to corresponding total energies and forces.^9–13^ These potentials are ML models typically based on neural networks^14–22^ or Gaussian process models.^3,13,23–25^ The advantage of ML potentials lies in their ability to perform molecular dynamics (MD) simulations with the accuracy of *ab initio* calculations but at only a fraction of the computational cost. As MD simulations often aim to explore the behavior and function of molecules and materials at the atomistic scale, constructing an initial dataset that fully represents all relevant geometries encountered during simulations is nearly impossible. Active learning approaches are therefore essential to update datasets and machine-learned potentials iteratively, ensuring both reliability and accuracy throughout the simulations.^24,26–38^ Typically, AL for machine-learned potentials is performed in a batch-wise manner, iterating through cycles of data labeling *via* quantum chemistry calculations, training ML models, and ML-accelerated simulations or other sampling strategies. Uncertainty quantification^25,39,40^ is often used to select the most informative samples for subsequent iterations (see Fig. 1a).

Despite the widespread use of AL for machine-learned potential development, the current infrastructures and algorithms often fail to fully utilize modern computational resources, relying heavily on human intervention for tasks such as labeled data handling and model training initialization. Furthermore, the lack of parallelism introduces inefficiencies, as AL is typically performed sequentially; for example, labeled data generation, model training, and exploration in molecular dynamics (MD) simulations are executed one after another. Due to the vastness of the target space commonly studied in scientific applications, this stepwise task execution of classic AL hinders both rapid iterative update of ML models and fast exploration of the interested space. Various studies have sought to address these limitations, for instance, by training models in parallel, accelerating data labeling using high-throughput computation within the oracle,^2,22,41–43^ or automating data flow through modularized task design.^44^ Nonetheless, these approaches typically demand complex implementations and continue to suffer from limited parallelism and inefficient data communication across different AL components.

To address these challenges, we developed PAL, an automated, modular, and parallel active learning workflow (see Fig. 1b) with several key advantages: (1) the fully automatic workflow minimizes human intervention during execution; (2) the modular and highly adaptive design reduces the effort of (re-)implementing parts of the active learning workflow while allowing it to be extended to various tasks with diverse combinations of resources, data, and ML model types; (3) the decoupling of all AL modules and use of surrogate models for exploration enables data and task parallelism, facilitating simultaneous exploration/generation, labeling, and training tasks; and (4) PAL is implemented using the Message Passing Interface (MPI) in Python,^45–47^ ensuring scalability and flexibility for deployment on both shared-memory systems (*e.g.*, laptops, local workstations) and distributed-memory systems (*e.g.*, high-performance computing clusters) with high data communication efficiency.

We demonstrate the applicability and versatility of PAL across various applications of machine-learned potentials and other machine learning tasks for science and engineering beyond atomistic simulations. Our results highlight the scalability of PAL and the significant speed-ups achieved through asynchronous parallelization on CPU and GPU hardware.

## 2 Description of the PAL workflow

**Fig. 2:**
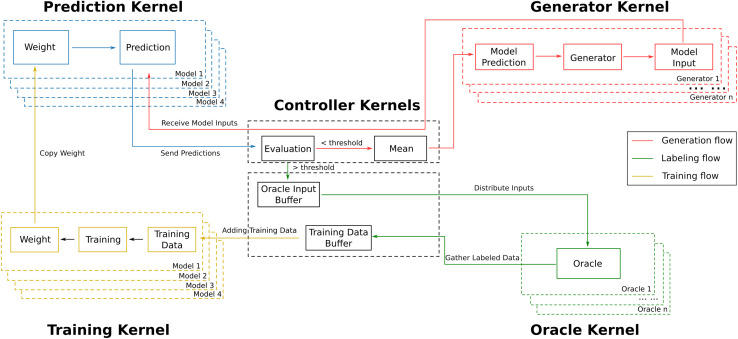
The computational architecture of the PAL workflow. Multiple boxes indicate parallelization of multiple instances of each kernel. The arrows illustrate information flow between the kernels orchestrated by the two controller sub-kernels. One dedicated controller sub-kernel ensures high-frequency communication between generation and prediction kernels.

PAL is designed as a modular and scalable framework that orchestrates the components of an active learning workflow in a parallel computing environment. The architecture of PAL centers around five core modules, referred to as kernels: the prediction kernel, generator kernel, training kernel, oracle kernel, and controller kernel (see Fig. 2). These kernels operate concurrently and communicate efficiently through the Message Passing Interface (MPI), enabling seamless integration and coordination on both shared- and distributed-memory systems.

At a high level, the prediction kernel provides machine learning models that make predictions on input data generated by the generators. The generator kernel explores the target space by producing new data instances based on the predictions received. The training kernel retrains the machine learning models using newly labeled data to improve their performance iteratively. The oracle kernel acts as the source of ground truth labels, providing accurate labels for selected data instances that require further clarification. Overseeing these components, the controller kernel manages the workflow by coordinating communication, scheduling tasks, and handling data exchanges between kernels.

This modular design allows users to customize and extend each kernel according to the specific requirements of their active learning scenarios. By decoupling the kernels and enabling them to operate in parallel, PAL maximizes computational resource utilization and minimizes overhead associated with sequential processing. The architecture supports asynchronous execution, where data generation, model prediction, labeling, and training can proceed simultaneously, leading to significant improvements in efficiency and scalability. A user-designated number of instances of the process in every kernel are generated during execution, each with distinct identifiers, data, and behaviors, tailored to the specific requirements of the kernel. The functionality of each kernel will be discussed in more detail in the subsequent sections.

### 2.1 Prediction kernel

In the prediction kernel, ML processes infer target values for inputs generated by the generators. Multiple ML models can operate concurrently when bootstrapping or query-by-committee techniques are employed. The controller aggregates their predictions and performs predefined or user-defined manipulations, such as calculating the mean and standard deviation, before distributing the results to generators and oracles. The separation of prediction and training tasks in PAL aims to minimize disruptions in data generation and inference caused by the time-consuming labeling and training processes. ML models in the prediction kernel are updated periodically by replicating weights from the corresponding models in the training kernel after a specified number of training epochs.

*Example*: in the case of machine-learned potentials, the prediction kernel is the machine learning model that predicts energies and forces given the system coordinates during an MD simulation or during other sampling methods used to explore the relevant geometry space (*e.g.* enhanced sampling methods or transition state search algorithms). Potential prediction kernels can be SchNet,^48^ Nequip,^14^ Allegro,^49^ MACE,^50^ and other machine-learned potentials.

### 2.2 Generator kernel

The generator kernel hosts an arbitrary number of generator processes running in parallel to accelerate data generation. Predictions from the ML models in the prediction kernel are disseminated to each generator by the controller, facilitating further data production. Each generator independently manages its data and maintains its generation–prediction-iteration status. Every generator can signal the controller kernel to shut down the PAL workflow upon meeting user-defined criteria.

*Example*: in the case of machine-learned potentials, this kernel is the exploration algorithm, *e.g.* a single MD step or the generation of a new geometry in the geometry-exploration method. The generator kernel communicates with the prediction kernel through the controller kernel to receive energy and force labels for given geometries, *e.g.* to propagate the system and propose new geometries. Neither the generator kernel nor the prediction kernel makes any decisions about the ML predictions' reliability, as the controller handles this process centrally. However, the generator kernel receives reliability information from the controller in order to decide whether to trust the energies and forces predicted by the ML models or whether to restart trajectories. That means that the uncertainty quantification and thus the decision of whether or not to label new geometries by the oracle is handled centrally by the controller kernel whereas the decision-making logic of how to react to uncertain predictions is implemented by the generator kernel. This offers flexibility and allows users to implement a wide range of workflows, *e.g.* allowing trajectories to propagate into regions of high uncertainty for a given number of steps (‘patience’).

### 2.3 Oracle kernel

The oracle kernel allows for deploying multiple oracle processes, each operating independently. Every oracle process keeps point-to-point communication with the controller, receiving input data for labeling. Once labeling is complete, the generated labels sets are returned to the controller before being distributed to the training kernel.

*Example*: in the case of machine-learned potentials, this kernel is the quantum chemical calculation that is used to generate the labels for the training data, *e.g.* density functional theory calculations to compute energies and forces of given input geometries. As described above, the decision about when to invoke additional DFT calculations to label data is performed centrally by the controller kernel. Once the active learning workflow is ‘converged’, *i.e.* the entire relevant input space is covered by the dataset and the uncertainty of the trained ML model on the final dataset does not reach a certain threshold value anymore, no new oracle calls will be requested anymore and PAL will run simulations by only iterating between generator and prediction kernels.

### 2.4 Training kernel

The training sets kept in the training kernel are expanded with new data labeled by oracles. An equal number of ML models as in the prediction kernel are trained in parallel within the training kernel, synchronizing with the labeling and prediction–generation processes. Training can be halted if a user-defined early stopping criterion is met to prevent overfitting, or restarted when new data points are introduced. For efficiency, trained model weights are periodically copied directly to the prediction kernel. The training kernel manages all model-related data, including scalars, hyperparameters, weights, and training histories, as models in the prediction kernel are considered replicas of those in the training kernel. Every process in the training kernel can signal the controller kernel to shut down the PAL workflow upon meeting user-defined criteria.

*Example*: in the case of machine-learned potentials, as discussed in the paragraph on prediction kernels above, the training kernel includes one epoch of training of any machine-learned potential, *e.g.* SchNet, Nequip, Allegro, or MACE, on a given data set. The user can define whether training should continue from the previous checkpoint or restart from a new random initialization after a certain number of epochs and active learning iterations.^51^ PAL offers full flexibility for the user. Generally, we recommend not to re-initialize weights during active learning which is also the default setting.

### 2.5 Controller kernel

The controller kernel orchestrates data communication, evaluates model predictions, selects inputs for labeling, and manages metadata storage (oracle input buffer and training data buffer). Data selected for labeling is buffered in the oracle input buffer and sent to the first available oracle. Labeled data is stored in the training data buffer and distributed to the ML models in the training kernel once the buffer size reaches a user-defined threshold. The data flow between the prediction and generator kernels is decoupled from the oracle and training kernels, ensuring efficient and uninterrupted communication between the prediction and generator kernels. The oracle and training kernels can be disabled to convert PAL into a prediction–generation workflow without significant performance impact, useful in scenarios where model training is unnecessary, such as ML-accelerated molecular dynamic simulations.

The user does not have to add any code to the controller kernel, except to provide functions that select instances for labeling and adjust the training data buffer dynamically (see Utilities in the ESI† for more detail). Other than that, the user only needs to specify and adjust the previous four kernels for prediction, generation, oracle, and training.

## 3 Illustrating applications

The PAL library developed and presented in this work has been applied in several scenarios in different application areas, also beyond atomistic simulations. The examples discussed in the following include photodynamics simulations using surface hopping algorithms based on neural network potentials and TD-DFT,^52^ hydrogen transfer reaction simulations in biological systems using graph neural network potentials trained on semiempirical methods as well as DFT,^53^ simulations of inorganic clusters using neural networks trained on DFT data, as well as surrogate machine learning models of fluid- and heat transport in textured channels trained on fluid dynamics simulation data.^54,55^ By altering the kernels (see Table 1), the library is adaptive to the different resource requirements, data structures, ground-truth oracles, and machine learning model types and architectures required by various scenarios.

**Table 1:** Summary of applications and corresponding kernel choices in PAL

Application	Prediction & training kernel	Generator kernel	Oracle kernel
Photodynamics simulations	Fully connected neural network committee	Parallel surface-hopping MD simulations	TDDFT (B3LYP/6-31G*) with Turbomole
HAT simulations	Graph neural network (SchNet, Allegro, MACE) committee	Randomized sampling of relevant geometries; transition state search	Semiempirical calculations with xTB and DFT (BMK/def2-TZVPD) with Turbomole
Inorganic clusters	Graph neural network (SchNet, MACE) committee	MD simulations with varying cluster sizes and charges	DFT (TPSS/dhf-TZVP) with Turbomole
Thermo-fluid flow optimization	Convolutional neural network committee	Particle swarm optimization	CFD simulations: in-house OpenFOAM solver

Due to the modularity of the PAL software architecture, generic code for each of the kernels can be customized by the user to accommodate the needs of the respective application areas. All generic technical aspects of communication are identical in all applications and thus transferrable. Clear interface definitions make it easy for users to implement custom kernels by integrating their own code in the user-defined kernel functions or by calling external software from the kernels. Users can contribute kernels or kernel blueprints to the PAL code, which will make it easier for future users to use those kernels and further customize them. Examples include different quantum chemistry codes as oracle kernels, different ML potentials for training and prediction kernels, and different molecular dynamics or enhanced sampling propagators as generators.

### 3.1 Photodynamics simulations

Organic semiconductors are essential materials in both emerging and commercially significant applications, such as organic solar cells and organic light-emitting diodes (OLEDs).^56^ While computational methods for predicting molecular properties using physics-based models^57^ as well as machine learning models^58–62^ are well developed, simulating complex phenomena like degradation remains challenging. This difficulty arises due to insufficient data to train machine learning (ML) models for these processes and the high computational cost of long excited-state dynamics simulations required by physical models.

**Fig. 3:**
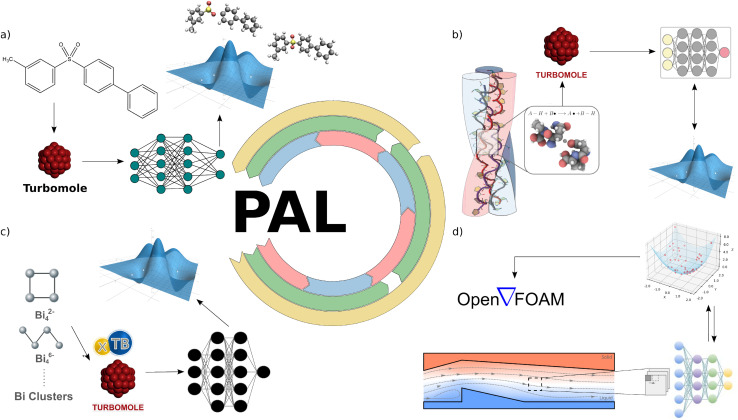
Examples of PAL applications. (a) Photodynamics simulations. (b) Hydrogen atom transfer reaction simulations. (c) Atomistic simulation of inorganic clusters. (d) Thermo-fluid flow properties optimization.

To address this challenge, we employ PAL to enable the simulation of multiple excited-state potential energy surfaces of a small molecule organic semiconductor, 3-methyl-4′-phenyl-diphenylsulfone. In this application of PAL, fully connected neural networks (NNs) are utilized in both the prediction and training kernels (see Fig. 3a). The prediction kernel leverages these NNs to approximate excited-state energies and forces efficiently.

For uncertainty quantification, we implement the query-by-committee method,^63^ where four NN models in the prediction kernel perform energy and force predictions for the same set of molecular geometry inputs generated by the generator kernel. The generator kernel runs 89 molecular dynamics simulations in parallel, each exploring different regions of the chemical space to discover unseen molecular geometries. The mean predictions from the committee are used to propagate the molecular dynamics trajectories.

When the standard deviation among the committee's predictions for a given geometry exceeds a predefined threshold, indicating high uncertainty, the corresponding geometries are forwarded to the oracle kernel. In the oracle kernel, accurate energy and force labels are computed using time-dependent density functional theory (TDDFT) at the B3LYP/6-31G* level of theory. These new data points are then added to the training set in the training kernel, where the NNs are retrained to improve their predictive accuracy.

We deployed this workflow on two hybrid CPU–GPU nodes of the HoreKa cluster. The forward pass of 89 geometries in parallel takes an average of 51.5 ms for each NN in the prediction kernel, while MPI communication and trajectory propagation require only 4.27 ms. Notably, removing the oracle and training kernels does not affect this result, indicating that the additional communication and data processing do not degrade the performance of the rate-limiting step.

By leveraging PAL, we achieve substantial computational savings compared to traditional sequential workflows. In this application, the TDDFT calculations are the computational bottleneck, which is reduced by PAL through parallelizing TDDFT across many CPU cores as well as running model training and MD simulations in parallel. Furthermore, avoiding similar and thus redundant TDDFT calculations, we benefit from the ability to run multiple molecular dynamics simulations in parallel, exploring different parts of the input space simultaneously and thus suggesting more diverse samples to be labeled by TDDFT. This accelerates the input space exploration and thus the development of reliable ML models for photodynamics simulations, facilitating the study of complex phenomena such as degradation in organic semiconductors.

### 3.2 Hydrogen atom transfer reaction simulations

Mechanical stress in collagen can lead to bond ruptures within the protein backbone, resulting in the formation of two radicals. These radicals, characterized by unpaired valence electrons, are highly reactive and can potentially cause damage to the surrounding biological environment. Hydrogen atom transfer (HAT) is a fundamental process in radical chemistry, wherein a hydrogen atom is abstracted from a donor molecule to an acceptor, generating a new radical species. Recent research on collagen has identified HAT processes as a critical mechanism for radical migration, playing a key role in mitigating damage caused by radicals produced through mechanical stress on collagen fibrils.^64^ Due to their short lifetimes, these radicals are challenging to observe experimentally, necessitating computational approaches for their study.

Traditional classical molecular dynamics (MD) simulations and even reactive force fields struggle to accurately describe radicals and HAT processes in proteins, primarily because they cannot adequately capture the quantum mechanical nature of bond breaking and formation. To overcome this limitation, we rely on machine-learned potentials to compute molecular energies and predict reaction barriers with high accuracy. This approach enables large-scale hybrid MD and kinetic Monte Carlo simulations, providing deeper insights into the mechanisms of radical migration and HAT reactions in biological systems.^65^

Constructing an effective training dataset for the machine-learned potentials presents several challenges. The potential energy surface (PES) of HAT reactions is complex, requiring sampling not only near-equilibrium configurations but also a diverse set of reaction pathways and transition states. Moreover, the trained model needs to generalize to unseen systems, such as different radical positions in unseen collagen environments, which necessitates training on various peptide combinations and reaction sites. Additionally, quantum chemical calculations to obtain ground truth energies and forces are computationally expensive, making efficient data generation and selection of informative training data essential.

In this application of PAL, the prediction kernel consists of a graph neural network (GNN). We utilize models such as SchNet,^48^ Allegro,^49^ and MACE,^50^ which have demonstrated high accuracy in predicting energies and forces for molecular systems. The GNN in the prediction kernel provides rapid inference of energies and forces for new configurations generated during simulations (see Fig. 3b).

The generator kernel employs a workflow that continuously generates new reaction data points. This is achieved through methods such as molecular dynamics simulations biased towards reaction coordinates or employing transition state search algorithms to explore possible reaction pathways. By generating a stream of diverse configurations, the generator kernel effectively samples the relevant configuration space for HAT reactions.

For labeling the most informative and uncertain configurations, the oracle kernel uses the quantum chemistry software Turbomole^66^ to perform calculations at the DFT/BMK/def2-TZVPD level of theory. These calculations provide accurate ground truth energies and forces, which are essential for refining the machine-learned potentials.

In the training kernel, we leverage pre-trained models as a starting point, incorporating information from previously generated initial datasets. The training kernel updates these models using the new labeled data through query-by-committee uncertainty quantification, to generalize better to unseen systems and reaction sites.

In this application example, we can generate an infinite stream of diverse unlabeled samples, moving the bottleneck to the labeling process by the oracle and the training process. Depending on the specific application, rather inexpensive oracles, *e.g.* xTB^67^ might be sufficient, shifting the bottleneck to the training. In such cases, by parallelizing all components within the PAL framework, we efficiently cover the relevant configuration space and benefit from a continuously updated ML model which is essentially trained on an infinite stream of newly generated data,^68^ preventing overfitting and ensuring optimal generalization. Here, this achieves accurate predictions of HAT reaction barriers with chemical accuracy. This approach accelerates the development of reliable machine-learned potentials for simulating complex biochemical reactions, ultimately enhancing our understanding of radical-mediated processes in biological systems.

### 3.3 Atomistic simulation of inorganic clusters

Clusters – groups of atoms or molecules held together in atomically precise geometries by chemical interactions – offer significant potential for creating new materials with tailored properties across diverse applications.^69^ Their unique characteristics distinguish them from both isolated molecules and bulk materials, presenting unique opportunities and challenges in computational modeling. Simulating inorganic clusters, particularly larger ones, is computationally demanding due to the necessity of accurate quantum mechanical calculations. This challenge is exacerbated for clusters containing heavy atoms like bismuth, where relativistic effects become significant and must be accounted for, further increasing computational complexity.

While ML-potentials have demonstrated the ability to predict energies and forces with high accuracy, most developments have focused on organic molecules and periodic materials. Organic molecules benefit from extensive datasets such as MD17,^70^ which facilitate the training of ML models. In contrast, datasets for inorganic clusters are virtually non-existent, making it difficult to train ML models or transfer knowledge from existing models.

To address this gap, we employ the PAL workflow to investigate the reactivity and transformations of small bismuth clusters (see Fig. 3c). Our goal is to demonstrate how ML-accelerated simulations can enhance our understanding of inorganic cluster formation and reactivity. In this application, we utilize graph neural networks as the prediction kernel, employing models like SchNet^48^ and MACE,^50^ which were originally designed for organic molecules.

We begin by pre-training these ML models on a foundational dataset of bismuth clusters to establish a baseline understanding of their behavior. Through the active learning workflow facilitated by PAL, we iteratively retrain the ML potentials on new configurations encountered during molecular dynamics (MD) simulations. The generator kernel produces MD trajectories for bismuth clusters of varying shapes, sizes, and charge states, effectively exploring the configurational space.

One significant challenge in modeling inorganic clusters is accounting for different charge states, as clusters can possess varying total charges leading to multiple potential energy surfaces. Reduction and oxidation processes involve interactions with environmental molecules, which are not explicitly modeled in this study. To address this, PAL's oracle kernel selectively labels highly uncertain and informative configurations using quantum mechanical calculations performed by Turbomole.^66^ These calculations provide accurate energies and forces for configurations that are poorly represented in the current training set.

The training kernel then incorporates these new data points to retrain the ML models, improving their accuracy and generalization to different charge states and cluster configurations. By dynamically updating the models, PAL enables them to capture complex information specific to bismuth cluster formation and their potential energy surfaces, extending beyond their original design focused on organic systems.

Similar to the first application, the bottleneck here is the labeling process in the oracle kernel, specifically for larger clusters. Additionally, as the ML model is not system-specific anymore, more complex ML potentials are used here, requiring flexibility and modularity to compare different ML potentials and potentially even move to periodic systems with explicit solvent molecules and counterions to even model redox-reaction events. Through the flexibility of PAL in combining different oracle and ML models, we effectively expand the scope of ML potentials to address the complexities inherent in inorganic clusters. This approach opens new avenues for simulating and understanding inorganic clusters, facilitating the development of materials with tailored properties.

### 3.4 Thermo-fluid flow properties optimization

As a final example of applying PAL, we move beyond the domain of atomistic modeling to demonstrate that the active learning workflows implemented in PAL are not limited to machine-learned potentials. Heat transfer in fluids is a complex process influenced by various factors such as geometry, material properties, and environmental conditions. While simple heat transfer problems can be solved analytically, most real-world scenarios require numerical simulations for accurate predictions. Computational fluid dynamics (CFD) is the primary method for numerically solving the governing equations of fluid mechanics and studying complex fluid flows without the need for physical experiments. However, high-fidelity CFD simulations are computationally expensive and time-consuming, despite providing high spatial and temporal resolution. This computational cost limits the ability to perform extensive parametric studies or real-time simulations.

To mitigate the high computational cost, machine learning models can be employed as surrogate models for CFD simulations, significantly reducing the overall computational cost while maintaining acceptable accuracy.^71–74^ In our application, the primary objective is to predict thermo-fluid flow properties – specifically, the drag coefficient (*C*_f_) and the Stanton number (St) – for two-dimensional laminar channel flows. Developing machine learning models capable of accurately predicting these fluid properties necessitates training datasets that encompass a wide variety of geometries and flow patterns. However, assembling such comprehensive datasets is challenging due to the computational expense of generating high-fidelity simulations for each configuration.

To address this challenge, we utilize PAL to strategically generate training data, thereby reducing the computational burden associated with simulating channel flows and leading to more efficient machine learning model development (see Fig. 3d). In this application of PAL: the prediction kernel consists of highly robust convolutional neural networks (CNNs) that are entirely invariant to image flipping and substantial shifting.^75^ These CNNs serve as surrogate models that predict *C*_f_ and St from input geometries, enabling rapid evaluations without the need for full CFD simulations. The generator kernel employs particle swarm optimization (PSO)^76^ to optimize the distribution of eddy-promoters within the flow domain.^54^ Eddy-promoters are geometric features introduced to enhance mixing and heat transfer. By optimizing their placement, we can explore a diverse set of flow configurations that are most informative for training the surrogate models. The oracle kernel utilizes an in-house developed OpenFOAM solver^77^ to perform high-fidelity CFD simulations. These simulations compute the flow and temperature fields, as well as the corresponding fluid flow properties *C*_f_ and St, providing accurate labels for the training data. The training kernel retrains the CNN models with the newly generated and labeled data, improving their predictive accuracy and generalization to new configurations. This iterative retraining ensures that the surrogate models remain accurate as they are exposed to new geometries and flow patterns.

Similar to the examples before, but in a very different application domain, there is not a unique bottleneck in any of the kernels but all kernels have similar computational costs. By integrating and parallelizing these components with PAL, we efficiently generate and select the most informative data for optimization while labeling them and training the ML surrogate model. Having the optimization process included in the generator allows us to focus computational resources on simulations that provide the greatest benefit not only for model performance but specifically for channel optimization, thereby reducing the total number of CFD simulations required to find good local or even the global optimum. As a result, we achieve surrogate models that can predict thermo-fluid flow properties of relevant close-to-optimal channel geometries with high accuracy while drastically reducing computational time compared to performing CFD simulations for every new configuration. This demonstrates PAL's versatility and effectiveness beyond atomistic simulations, highlighting its potential for applications in engineering domains where computational cost is a limiting factor. By leveraging PAL, we can perform rapid optimization and design studies in thermo-fluid systems, contributing to advancements in fields such as heat exchanger design, microfluidics, and aerodynamic surface optimization.

## 4 Discussion: library use, current limitation, and future developments

### 4.1 Using PAL to customize and automate active learning workflows

With increasing popularity of ML potentials, not only in proof-of-principle studies but also in exploratory research of novel systems, also the role of active learning increases, and thus the need for efficient, customizable, and easy-to-use implementations. While different application scenarios have different requirements on the specific methods used in active learning, *i.e.* the ML models and the data generation algorithms and methods, the general workflow and the required communication backbone is the same in almost all cases. Thus, this backbone does not need to be implemented by every researcher or research group. Furthermore, in the spirit of open software, specific parts and modules in active learning workflows should be shared by users to make it easier in the future to build new active learning workflows. Our parallel active learning library PAL is expected to be used by researchers to integrate, customize, and develop different parts of active learning workflows without having to reimplement the entire communication and workflow scheduling backbone again and again. PAL helps to automate active learning workflow while minimizing the effort of modifications in the implementation. Thus, we believe it can help significantly enhance the efficiency of constructing training sets, training ML potentials, and applying them to interesting systems.

The parallel active learning workflow presents a substantial improvement over traditional serial approaches by effectively utilizing high-performance computing resources. Please refer to the ESI† for detailed speedup estimation with several common application specifications, *e.g.* molecular dynamics with a DFT oracle, reaction network exploration with semiempirical oracle, and computational fluid dynamics with particle swarm optimization as the explosive generator.

The current source code of PAL includes the general and generic backbone for communication and workflow automation as well as blueprints/placeholders of the different application-specific kernels – ML potential training and prediction, oracle, and generator. We furthermore provide example implementations of those modules for the four application examples discussed in Section 3. Those can easily be used, mixed, and adapted by users. We plan to develop more prototypical kernels in the future which will make it easier for users to combine them to active learning workflows with minimal coding effort. We also encourage users to contribute additional kernels to the library, to make them accessible to other researchers.

### 4.2 Hardware

The current PAL workflow is implemented and tested on the Slurm workload manager, using a single type of computational node (CPU node, GPU node, or CPU–GPU hybrid node). Executing PAL on other scheduling systems will require additional user input to specify computational resources. We plan to extend this work to support additional batch systems and enhance flexibility in node scheduling, job assignment, and resource management. Another future goal is to incorporate real-time tracking and monitoring of timing and resource usage, such as GPU and memory utilization, to facilitate workflow optimization in various scenarios.

### 4.3 Communication bottleneck

When the inference time of ML models in the prediction kernel is 10 ms or less, communication between the generator and predictor can become a bottleneck limiting the speed of exploration in the generator. Solving this requires further work to couple generator and prediction kernel more tightly, though this is not currently an issue for typical ML potentials, especially complex models such as equivariant graph neural networks. Additionally, if the shape of the ML input or output is not fixed, there is an overhead as MPI messages require a predetermined size to be efficient.

### 4.4 Available kernels

As discussed in Section 3 and above, the PAL library currently includes a few examples of generators, ML models, and oracles, primarily in the fields of materials science, chemistry, and engineering. We plan to expand these examples to encompass more relevant applications of active learning in the future.

## 5 Conclusion

In this work, we introduced PAL, an automated, modular, and parallel active learning library designed to overcome the limitations of traditional active learning workflows that often require extensive human intervention and underutilize modern computational resources. By decomposing the active learning process into five modular core kernels, the prediction kernel, generator kernel, training kernel, oracle kernel, and controller kernel, PAL enables asynchronous and parallel execution of data generation, labeling, model training, and prediction tasks. This modular architecture allows users to easily customize and extend each component to suit a wide range of applications across different scientific and engineering domains.

Our examples illustrate how PAL significantly reduces computational overhead and improves scalability, achieving substantial speed-ups through asynchronous parallelization on both CPU and GPU hardware. By decoupling the prediction and training processes, PAL minimizes disruptions caused by time-consuming labeling and model updates, ensuring efficient utilization of high-performance computing resources. The library's flexibility and effectiveness are showcased through its successful application to diverse real-world scenarios, including photodynamics simulations of organic semiconductors, hydrogen atom transfer reactions in biological systems, atomistic simulations of inorganic clusters, and thermo-fluid flow optimization in engineered systems.

PAL advances the field of scientific active learning by providing a scalable and adaptable framework that streamlines the integration of machine learning models, uncertainty estimation methods, oracles, and data exploration strategies. It facilitates the development of highly accurate models with minimal data acquisition costs, thereby accelerating research and discovery in various domains. The ability to handle complex workflows and large-scale computations makes PAL a valuable tool for scientists and engineers seeking to leverage active learning in their work.

Looking ahead, future developments of PAL will focus on enhancing its capabilities and user experience. Plans include supporting additional batch systems HPC environments, incorporating real-time monitoring and resource management features, and integrating with other machine learning frameworks and tools. We also aim to expand the library's documentation and provide comprehensive tutorials to lower the adoption barrier for new users. By addressing current limitations and fostering community contributions, we hope that PAL becomes a useful and widely applied tool in active learning workflows of ML potentials and beyond, empowering researchers to efficiently harness computational resources and drive innovation in their respective fields.

## Author contributions

P. F. and C. Z. contributed to the conceptualization of the project. C. Z. is the main developer of the methodology and software. M. N., Y. K., and Y. Z. contributed aspects of the software. P. F. contributed resources, supervision, and funding acquisition. All authors contributed to writing the original draft as well as to visualization of the content.

## Conflicts of interest

There are no conflicts of interest to declare.

## Supplementary Material

DD-004-D5DD00073D-s001

DD-004-D5DD00073D-s002

## Data Availability

The code for PAL is available through the open-source repository on GitHub for continuous development, also by the community: https://github.com/aimat-lab/PAL (https://doi.org/10.5281/zenodo.15658962). The version of the code employed for this study is version 2.1.1. The hydrogen atom transfer reaction simulations case study described in Section 3.2 was carried out using publicly available data from heiData at https://doi.org/10.11588/DATA/TGDD4Y (https://doi.org/10.26434/chemrxiv-2023-7hntk). The case study of thermo-fluid flow properties optimization described in Section 3.4 was carried out using publicly available data from GitHub at https://github.com/aimat-lab/ChemEngML (https://doi.org/10.1063/5.0187783). The data and models supporting the application use cases discussed in this work can be found in the individual publications.^75,78,79^
