# Supplementary figures and images for: PAL – parallel active learning for machine-learned potentials
Source: Digit Discov. 2025 Jun 22;4(7):1901–11. doi: 10.1039/d5dd00073d (PMC12188519; doi:10.1039/d5dd00073d)

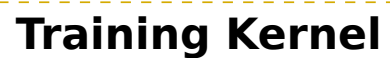

Supplement: DD-004-D5DD00073D-s001 [file DD-004-D5DD00073D-s001.pdf]
