# Supplementary material for: PAL – parallel active learning for machine-learned potentials
Source: Digit Discov. 2025 Jun 22;4(7):1901–11. doi: 10.1039/d5dd00073d (PMC12188519; doi:10.1039/d5dd00073d)
Supplement: DD-004-D5DD00073D-s002 [file DD-004-D5DD00073D-s002.pdf]

Supplementary Information (SI) for Digital Discovery.
This journal is © The Royal Society of Chemistry 2025

PAL - Parallel active learning for machine-learned potentials

Chen Zhou[1,2], Marlen Neubert[1,2], Yuri Koide[1,2], Yumeng Zhang[1,2], Van-Quan Vuong[1,2,3], Tobias Schlöder[1,2], Stefanie Dehnen[2], and Pascal Friederich[1,2,*]

[1]Institute of Theoretical Informatics, Karlsruhe Institute of Technology, Kaiserstr. 12, 76131 Karlsruhe, Germany
[2]Institute of Nanotechnology, Karlsruhe Institute of Technology, Kaiserstr. 12, 76131 Karlsruhe, Germany
[3]Institute for Physical Chemistry, Karlsruhe Institute of Technology, Kaiserstr. 12, 76131 Karlsruhe, Germany
[*]Corresponding author: pascal.friederich@kit.edu

Supporting information

S1 Parallelization with MPI

The PAL is designed in an object-oriented manner, wherein the implementation of each kernel is for a single corresponding process and the user-specified number of processes are generated for every kernel during execution. The methods of different processes and data flow among them are displayed in Figure 1. Each process is assigned a unique ID (MPI rank) and maintains its data, states, and behaviors. For communication efficiency, data transferred among kernels should be arranged as 1-D Numpy numerical arrays. The communication processes are handled by the Controller kernel separated from the user with MPI message-passing methods, including broadcast, gather, scatter, and point-to-point communications. Explanations of every kernel are presented in the following sections with a toy example in which random numbers are generated in Generator and Oracle kernels and sent to Prediction and Training kernels for inference and training.

S2 Speedup estimation for parallel active learning workflow

In this section, we present a detailed estimation of the speedup achievable by the Parallel Active Learning (PAL) workflow compared to a fully serial active learning workflow. We define the relevant parameters, derive the runtime equations for both workflows, and provide speedup estimates for specific use cases.

S2.1 Relevant parameters, runtimes, and speedup

To estimate the runtime and speedup, we consider the following parameters in a simplified setting:

- t_{oracle}: Time to label a single sample using the oracle.
- t_{train}: Time to train the machine learning (ML) model.
- t_{gen}: Time to perform 1000 steps of the generator and predictor modules.
- N: Number of samples to be labeled.
- P: Number of parallel workers available for labeling. We always assume that $P \leq N$

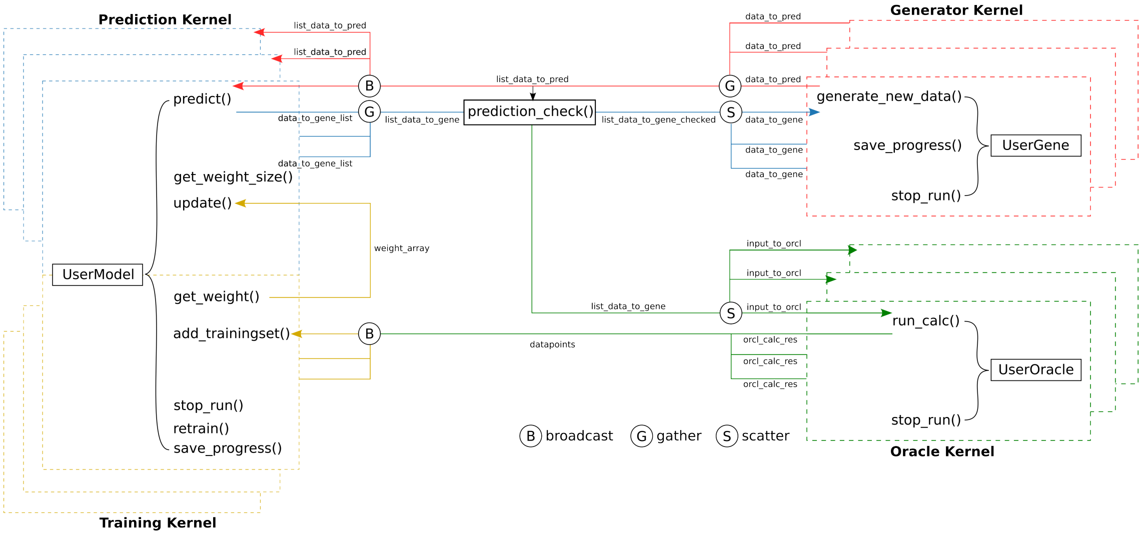

Figure 1: Methods and data flow of PAL.

Rumtime of the serial workflow

In the serial active learning workflow, assuming only parallelization of the oracles, the processes are executed sequentially in the following order: labeling, training, generation/prediction. The total runtime for N samples is given by:

$$T_{\text{serial}} = \frac{N}{P} \cdot t_{\text{oracle}} + t_{\text{train}} + t_{\text{gen}} \tag{1}$$

Rumtime of the parallel workflow

In the PAL workflow, the labeling, training, generation, and prediction modules operate in parallel, coordinated by the central module. Assuming perfect parallelization and ignoring communication overhead, the runtime can be approximated by the maximum of the individual module runtimes:

$$T_{\text{parallel}} = \max\left(\frac{N}{P} \cdot t_{\text{oracle}}, t_{\text{train}}, t_{\text{gen}}\right) \tag{2}$$

Speedup

The speedup (S) achieved by the PAL workflow over the serial workflow is defined as:

$$S = \frac{T_{\text{serial}}}{T_{\text{parallel}}} \tag{3}$$

Substituting the expressions for T_{serial} and T_{parallel}, we obtain:

$$S = \frac{\frac{N}{P} \cdot t_{\text{oracle}} + t_{\text{train}} + t_{\text{gen}}}{\max\left(\frac{N}{P} \cdot t_{\text{oracle}}, t_{\text{train}}, t_{\text{gen}}\right)} \tag{4}$$

Note that this speedup is only a lower bound of the speedup, as in the parallel workflow, all available resources are used and never idle. Thus, if the oracle is the bottleneck, the training and the generation/prediction tasks are continued and thus train over more epochs and explore more parts of the input space than the serial version of the workflow.

S2.2 Use case specific estimates

We consider three distinct use cases with varying oracle methods, ML models, and generators. For each use case, we estimate the speedup based on the provided parameters.

Use Case 1: DFT and GNNs

- Oracle: DFT, $t_{\text{oracle}} \approx 1$ hour
- ML Model: Graph Neural Network (GNN), $t_{\text{train}} \approx 1$ hour
- Generator: Molecular Dynamics (MD), $t_{\text{gen}} \ll 1$ hour

Assuming P parallel workers:

$$T_{\text{serial}} = \frac{N}{P} \cdot t_{\text{oracle}} + t_{\text{train}} + t_{\text{gen}} \approx \frac{N}{P} \cdot t_{\text{oracle}} + t_{\text{train}} \tag{5}$$

$$T_{\text{parallel}} = \max\left(\frac{N}{P} \cdot t_{\text{oracle}}, t_{\text{train}}, t_{\text{train}}\right) = \max\left(\frac{N}{P} \cdot t_{\text{oracle}}, t_{\text{train}}\right) \tag{6}$$

Thus, the speedup is:
In case that oracle evaluations and model training require similar computation times ($t_{\text{oracle}} = t_{\text{train}} = t$), and if we assume $N \geq P$ (leading to $T_{\text{parallel}} = \frac{N}{P} \cdot t_{\text{oracle}}$), the speedup obtained through PAL is

$$S = \frac{\frac{N}{P} \cdot t + t}{\frac{N}{P} \cdot t} = \frac{\frac{N}{P} + 1}{\frac{N}{P}} = \left(1 + \frac{P}{N}\right) \tag{7}$$

This means that in the case of balanced costs between oracle and training modules, the highest speedup is obtained if the number of parallel workers for labeling P is equal to the number of oracle calls N per iteration. This ratio is automatically optimized by PAL through asynchronous communication, leading to a speedup of 2 compared to serial active learning workflows.
However, if DFT calculations are much more time-consuming than model training, parallelization through a large number of parallel oracle calculations P is the limiting factor, indicating that best results are achieved if P in maximized.

Use Case 2: Semiempirical oracle for reaction network exploration

- Oracle: xTB, $t_{\text{oracle}} = 10$ seconds
- ML Model: Graph Neural Network (GNN), $t_{\text{train}} = 1$ hour
- Generator: Transition state search, $t_{\text{gen}} = 10$ minutes

In this case, the bottleneck is clearly the model training, as during training time, $360P$ new calculations can be done by the oracle. With a clear bottleneck, the parallel and serial runtime are approximately the same, leading to no substantial speedup through parallelization:

$$T_{\text{serial}} \approx t_{\text{train}} \tag{8}$$

$$T_{\text{parallel}} \approx \max\left(t_{\text{oracle}} + t_{\text{train}} + t_{\text{gen}}\right) = t_{\text{train}} \tag{9}$$

$$S \approx \frac{t_{\text{train}}}{t_{\text{train}}} = 1 \tag{10}$$

In this particular case, we recommend using a rolling training set where newly incoming xTB-labeled samples are added after every single training epoch, and old samples are removed to keep the training set size constant. This limits the training time per epoch and at the same time avoids overfitting to a fixed training set, ensuring permanent domain adaptation to the relevant molecules and geometries in the region of the chemical space that is currently explored by the generator. This dynamic and asynchronous way how data is communicated between modules in PAL makes this easily possible without users to write dedicated code for communication and scheduling.

Use Case 3: Computational fluid dynamics (CFD)

- Oracle: CFD, $t_{\text{oracle}} = 10$ minutes
- ML Model: Convolutional Neural Network (CNN), $t_{\text{train}} = 10$ minutes
- Generator: Particle Swarm Optimization (PSO), $t_{\text{gen}} = 10$ minutes

In this scenario, assuming $P = N$ parallel workers, and defining $t_{\text{oracle}} = t_{\text{train}} = t_{\text{gen}} = t$, all three modules have balanced costs, leading to the following runtimes:

$$T_{\text{serial}} = t_{\text{oracle}} + t_{\text{train}} + t_{\text{gen}} = 3t \tag{11}$$
$$T_{\text{parallel}} = \max\left(t_{\text{oracle}} + t_{\text{train}} + t_{\text{gen}}\right) = t \tag{12}$$

Thus, the speedup is:

$$S = \frac{3t}{t} = 3 \tag{13}$$

Thus, in scenarios without a clear bottleneck, i.e. where the computational cost of all modules is similar, the speedup approaches 3, independent of other considerations. Thus, when designing parallel active learning workflows with PAL, it is desirable to break down the computational steps in all modules into small pieces (one single oracle call, potentially with sub-parallelization, one single epoch of training, and one short generator run to explore the input space), allowing load-balancing and thus optimal parallelization for high speedup factors.

S2.3 Conclusion

The Parallel Active Learning workflow presents a substantial improvement over traditional serial approaches by effectively utilizing high-performance computing resources. The speedup estimations for various use cases highlight PAL's versatility and efficiency, making it a valuable tool for researchers in computational science and beyond.

S3 PAL settings

Settings of a toy PAL example are displayed in the code block below. The user defines the number of processes in each kernel by setting `pred_process`, `orcl_process`, `gene_process`, and `ml_process`. The total number of processes initialized by PAL should be the summation of processes in the four kernels with two additional processes for the Controller. The user can also specify the distribution of processes among computational nodes by setting `designate_task_number` as `True` and designating the distribution in `task_per_node`. As the MPI requires to know the message size at the receiving end before communication, `fixed_size_data` should be set to `False` if inputs from Generator processes or predictions from Prediction processes vary in size for different data points, in which case sizes of data are passed first for every MPI communication.

```
AL_SETTING = {
    "result_dir": '../results/TestRun',    # directory to save all metadata and results
    "orcl_buffer_path": '../results/TestRun/ml_buffer',    # path to save data ready to send to
      ML. Set to None to skip buffer backup.
    "ml_buffer_path": '../results/TestRun/orcl_buffer',    # path to save data ready to send to
      Oracle. Set to None to skip buffer backup.

    # Number of process in total = 2 MPI communication processes (Manager and Exchange)
    #                              + pred_process + orcl_process + gene_process + ml_process
    "pred_process": 3,                     # number of prediction processes
    "orcl_process": 5,                     # number of oracle processes
    "gene_process": 20,                    # number of generator processes
    "ml_process": 3,                       # number of machine learning processes
    "designate_task_number": True,         # set to True if need to specify the number of tasks
      running on each node (e.g. number of model per computation node)
                                           # if False, tasks are arranged randomly
    "fixed_size_data": True,              # set to True if data communicated among kernels have
      fixed sizes.
                                           # if false, additional communications are necessary
     for each iteration to exchange data size info thus lower efficiency.
    "task_per_node":{                      # designate the number of tasks per node, used only
     if designate_task_number is True
        "prediction": [3, 0],              # list for the number of tasks per node (length must
      matches the number of nodes), None for no limit
        "generator": None,                 # list for the number of tasks per node (length must
      matches the number of nodes), None for no limit
        "oracle": None,                    # list for the number of tasks per node (length must
      matches the number of nodes), None for no limit
        "learning": [0, 3],                # list for the number of tasks per node (length must
      matches the number of nodes), None for no limit
    },
    "orcl_time": 10,                       # Oracle calculation time in seconds
    "progress_save_interval": 60,          # time interval (in seconds) to save the progress
```

```
    "retrain_size": 20,                    # batch size of increment retraining set
    "dynamic_orcale_list": True,           # adjust data points for orcale calculation based on
      ML predictions everytime when retrainings finish
    "gpu_pred": [],                        # gpu index list for prediction processes
    "gpu_ml": [],                          # gpu index list for machine learning
    "usr_pkg": {                           # dictionary of paths to user implemented modules (
     generator, model, oracle and utils)
        "generator": "../usr_example/generator.py",
        "model": "../usr_example/model.py",
        "oracle": "../usr_example/oracle.py",
        "utils": "../usr_example/utils.py",
    },
    }
```

S4 Prediction kernel

This kernel is implemented together with the Training kernel and behaviors differently from the latter by calling different methods during execution. The initialization of a prediction process is shown in the code below where the process ID (PID or rank), path to the working directory (`result_dir`), and device index (`i_device`) are assigned by the Controller.

```
def __init__(self, rank, result_dir, i_device, mode):
    """
    Initilize the model.

    Args:
        rank (int): current process rank (PID).
        result_dir (str): path to directory to save metadata and results.
        i_device (int): Index for assigning computation to device (GPU or CPU).
        mode (str): 'predict' for Prediction and 'train' for Machine Learning.
    """
    self.rank = rank
    self.result_dir = result_dir
    self.mode = mode
    self.i_device = i_device

    if self.mode == "predict":
        self.model = TestModel(4, 4)
        self.para_keys = list(self.model.state_dict().keys())

    else:
        ... ...
```

For inference, the Prediction process takes a list of 1-D arrays (`list_data_to_pred`) that consists of output values (`data_to_pred`) of `generate_new_data` method of Generator processes gathered by the Controller (Figure 1, flow in red). In cases where multiple Prediction processes are running in parallel, the same copy of `list_data_to_pred` is broadcasted to each process. The prediction method handles the organization of arguments, prediction of target values, and organization of values returning to the Generator kernel. The prediction method should return a list of 1-D arrays (`data_to_gene_list`) that are distributed to each process in the Generator kernel, thus the size of `data_to_gene_list` and the order of 1-D arrays should match processes in Generator kernel. The `data_to_gene_list` returned by every Prediction process are gathered by the Controller and passed through the user-defined `prediction_check` function before scattering to Generator processes' `generate_new_data` method (Figure 1, flow in blue). If the PAL workflow is shutdown by any Training process or Generator process, the `stop_run` method is called by every Prediction process before quitting.

```
def predict(self, list_data_to_pred):
    """
    Make prediction for list of inputs from Generator.

    Args:
        list_data_to_pred (list): list of user defined model inputs. [1-D numpy.ndarray, 1-D
     numpy.ndarray, ...]
                           size is equal to number of generator processes
                           Source: list of data_to_pred from UserModel.generate_new_data().

    Returns:
        data_to_gene_list (list): predictions returned to Generator. [1-D numpy.ndarray, 1-D
     numpy.ndarray, ...]
                                  size should be equal to number of generator processes
                                  Destination: list of data_to_gene at UserModel.
     generate_new_data().
    """
    data_to_gene_list = None
```

```python
    ##### User Part #####
    # organize the input data into a ndarray
    input_array = np.array(list_data_to_pred, dtype=float)

    self.model.eval()
    with torch.no_grad():
        x = torch.tensor(input_array, dtype=torch.float)
        #print(f"Debug Rank {self.rank}: data_to_gene shape {x.shape}")
        data_to_gene_list = list(self.model(x).numpy())

    # data_to_gene_list should be a list containing 1-D numpy arrays
    return data_to_gene_list

def stop_run(self):
    """
    Called before the Prediction/Training process terminating when active learning workflow
     shuts down.
    """
    ##### User Part #####
    if self.mode == "train":
        self.save_progress()
```

The ML model in each Prediction process is updated by replicating weight parameters from the corresponding model in the Training kernel, where the weights are arranged as a 1-D array (`weight_array`) by the Training process `get_weight` method and sent to the Prediction process directly. The Prediction process then calls the `update` method to reorganize the weight array and update the ML model. As the MPI requires to know the message size at the receiving end before communication, the `get_weight_size` method is called once when PAL workflow is initialized to return the size of the 1-D `weight_array`.

```python
def update(self, weight_array):
    """
    Update model/scalar with new weights in weight_array.

    Args:
        weight_array (1-D numpy.ndarray): 1-D numpy array containing model/scalar weights.
                                          Source: weight_array from UserModel.get_weight().
    """
    ##### User Part #####
    for k in self.para_keys:
        self.model.state_dict()[k] = weight_array[:self.model.state_dict()[k].flatten().shape
     [0]].reshape(self.model.state_dict()[k].shape)
    print(f"Prediction Rank {self.rank}: model updated")

def get_weight_size(self):
    """
    Return the size of model weight when unpacked as an 1-D numpy array.
    Used to send/receive weights through MPI.

    Returns:
        weight_size (int): size of model weight when unpacked as an 1-D numpy array.
    """
    weight_size = None

    ##### User Part #####
    weight_size = 0
    for k in self.para_keys:
        weight_size += self.model.state_dict()[k].flatten().shape[0]

    # weight_size should be returned as an integer
    return weight_size
```

S5 Training kernel

The Training kernel is implemented together with the Prediction kernel and distinguished from the former by the `mode` flag. Other than training/retraining ML models, Training processes are also responsible for saving data of interest, such as training history and model parameters. This is achieved by the `save_progress` method that is called whenever the training stops or restarts.

```python
def __init__(self, rank, result_dir, i_device, mode):
    """
    Initize the model.

    Args:
        rank (int): current process rank (PID).
        result_dir (str): path to directory to save metadata and results.
        i_device (int): Index for assigning computation to device (GPU or CPU).
        mode (str): 'predict' for Prediction and 'train' for Machine Learning.
    """
    self.rank = rank
```

```
    self.result_dir = result_dir
    self.mode = mode
    self.i_device = i_device

    if self.mode == "predict":
        ... ...

    else:
        self.start_time = time.time()
        self.x_train = []
        self.y_train = []
        self.x_val = []
        self.y_val = []
        self.val_split = 0.2
        self.model = TestModel(4, 4)
        self.para_keys = list(self.model.state_dict().keys())
        self.loss = nn.MSELoss(reduction='sum')
        self.optimizer = torch.optim.Adam(self.model.parameters(), lr=1e-3)
        self.max_epo = 1000000
        self.batch_size = 10
        self.history = {
            "MSE_train": [],
            "MSE_val": []
            }

def save_progress(self):
    """
    Save the current progress/data/state.
    Called everytime after retraining and receiving new data points.
    """
    ##### User Part #####
    with open(os.path.join(self.result_dir, f"retrain_history_{self.rank}.json"), 'w') as fh:
        json.dump(self.history, fh)
```

Once the user-defined number of new data are collected by the Controller from the Oracle kernel, the data collection is broadcasted to each Training process as a list of lists (`datapoints`). Each sub-list contains two 1-D arrays with the first one being the input and the second being label. The Prediction processes call `add_trainingset` to organize the new data and add to the training/validation set (Figure 1, flow in yellow).

```
def add_trainingset(self, datapoints):
    """
    Increase the training set with set of data points.

    Args:
        datapoints (list): list of new training datapoints.
                           Format: [[input1 (1-D numpy.ndarray), target1 (1-D numpy.ndarray)],
     [input2 (1-D numpy.ndarray), target2 (1-D numpy.ndarray)], ...]
                           Source: input_for_orcl element of input_to_orcl_list from utils.
     prediction_check().
                                   orcl_calc_res from UserModel.run_calc().
    """
    ##### User Part #####
    idx = np.array(range(0, len(datapoints)), dtype=int)
    val_size = int(self.val_split * idx.shape[0])
    i_val = np.random.choice(idx, size=val_size, replace=False)
    i_train = np.array([i for i in idx if i not in i_val], dtype=int)
    for i in range(0, len(datapoints)):
        if i in i_train:
            self.x_train.append(datapoints[i][0])
            self.y_train.append(datapoints[i][1])
        else:
            self.x_val.append(datapoints[i][0])
            self.y_val.append(datapoints[i][1])
    print(f"Training Rank{self.rank}: training set size increased")
```

The Training processes keep training/retraining ML models, which can be halted by a): new training data arriving or b): a user-defined early stopping criterion met, where a) is achieved by checking `req_data.Test()` at every training epoch and stops when the return value is `True`. The entire PAL workflow can be shut down by any one of the Training processes if the return flag `stop_run` is set to `True`, in which scenario each Training process calls `stop_run` method before quitting. Otherwise, the process is halted until new training data arrive and the retraining restarts by repeatedly calling the `retrain` method.

```
def retrain(self, req_data):
    """
    Retrain the model with current training set.
    Retraining should stop before or when receiving new data points.

    Args:
        req_data (MPI.Request): MPI request object indicating status of receiving new data
     points.
```

```
    Returns:
        stop_run (bool): flag to stop the active learning workflow. True for stop.
    """
    stop_run = False
    ##### User Part #####
    print(f"Training Rank{self.rank}: retraining start...")
    for v in self.history.values():
        v.append([])
    x_t = torch.tensor(np.array(self.x_train, dtype=float), dtype=torch.float)
    y_t = torch.tensor(np.array(self.y_train, dtype=float), dtype=torch.float)
    x_v = torch.tensor(np.array(self.x_val, dtype=float), dtype=torch.float)
    y_v = torch.tensor(np.array(self.y_val, dtype=float), dtype=torch.float)
    n_batch = int(len(self.x_train) / self.batch_size)
    n_batch_val = int(len(self.x_val) / self.batch_size)
    for i in range(1, self.max_epo+1):
        self.model.train()
        mse = 0
        for j in range(1, n_batch+1):
            pred = self.model(x_t[j*self.batch_size:min((j+1)*self.batch_size, x_t.shape[0])])
            loss = self.loss(pred, y_t[j*self.batch_size:min((j+1)*self.batch_size, y_t.shape
    [0])])

            loss.backward()
            self.optimizer.step()
            self.optimizer.zero_grad()
            mse += loss.item()
        self.history["MSE_train"][-1].append(mse/x_t.shape[0])
        if i % 10 == 0:
            self.model.eval()
            mse = 0
            for j in range(1, n_batch_val+1):
                with torch.no_grad():
                    pred = self.model(x_v[j*self.batch_size:min((j+1)*self.batch_size, x_v.
    shape[0])])
                    loss = self.loss(pred, y_v[j*self.batch_size:min((j+1)*self.batch_size, y_v
    .shape[0])]).item()
                    mse += loss
            self.history["MSE_val"][-1].append(mse/x_v.shape[0])

        # req_data.Test() indicates if new data have arrived from Oracle through MG
        if req_data.Test():
            # if new data arrive, stop retraining to update training/validation set
            print(f"Training Rank{self.rank}: new data arrive.")
            break
    print(f"Training Rank{self.rank}: retraining stop.")
    if time.time() - self.start_time >= 3600:
        stop_run = True
        print(f"Training Rank{self.rank}: stop signal sent.")
    else:
        stop_run = False

    # stop_run should be returned as a bool value.
    return stop_run

def stop_run(self):
    """
    Called before the Prediction/Training process terminating when active learning workflow
     shuts down.
    """
    ##### User Part #####
    if self.mode == "train":
        self.save_progress()
```

S6 Generator kernel

Processes in the Generator kernel are generated with its unique rank and working directory (`result_dir`). Each Generator process keeps its own records of state and data such as previously generated data or the number of inference-generation iterations, and saves records to `result_dir` through `save_progress` method after a user-defined interval (`progress_save_interval` in `al_setting`). Processes in the Generator kernel are alive through the entire PAL lifetime. Killing and restarting a Generator process is impossible in current PAL, while similar behavior could be achieved in the `generate_new_data` method.

```
def __init__(self, rank, result_dir):
    """
    initilize the generator.

    Args:
        rank (int): current process rank (PID).
        result_dir (str): path to directory to save metadata and results.
```

```python
        """
        self.rank = rank
        self.result_dir = result_dir
        ##### User Part ######
        self.counter = 0
        #self.limit = float("inf")
        self.limit = 300000 + self.rank
        self.state = np.random.randn(4)
        self.history = [[],]
        self.save_path = os.path.join(self.result_dir, f"generator_data_{rank}")

    def save_progress(self):
        """
        Save the current state and progress. Called everytime after the interval defined by
         progress_save_interval in al_setting.
        """
        ##### User Part #####
        m = 'ab' if os.path.exists(self.save_path) else 'wb'
        with open(self.save_path, m) as fh:
            pickle.dump(self.history[:-1], fh)
        self.history = self.history[-1:]
```

The Generator processes call `generate_new_data` method to generate new data and organize them as 1-D arrays (`data_to_pred`) that are gathered by Controller and broadcasted to Prediction processes for inference (Figure 1, flow in red). The `generate_new_data` method takes `data_to_gene` as input, which contains `None` value at the first AL iteration and predictions from the Prediction kernel through the `prediction_check` function for the rest. The entire PAL workflow can be shut down by any one of the Generator processes if the return flag `stop_run` is set to `True`, in which scenario each Generator process calls `stop_run` method before quitting.

```python
    def generate_new_data(self, data_to_gene):
        """
        Generate new data point based on data_to_gene (prediction from Prediction kernel).

        Args:
            data_to_gene (1-D numpy.ndarray or None): data from prediction kernel through EXCHANGE
         process.
                                                      Initialized as None for the first time step.
                                                      Source: element of data_to_gene_list from
         UserModel.predict()

        Returns:
            stop_run (bool): flag to stop the active learning workflow. True for stop.
            data_to_pred (1-D numpy.ndarray): data to prediction kernel through EXCHANGE process.
                                              Destination: element of input_list at UserModel.
         predict()
        """
        stop_run = False
        data_to_pred = None

        # please notice that data_to_gene is intinilized to be None for the first iteration.
        ##### User Part #####
        # in this simple example, generator processes return random numbers
        if self.counter > self.limit:
            stop_run = True
            data_to_pred = np.random.randn(4)
            print(f"Generator rank{self.rank}: stop signal sent.")
        elif data_to_gene is None:
            data_to_pred = np.random.randn(4)
            self.history.append([data_to_pred,])
        elif (data_to_gene == 0).any():
            data_to_pred = np.random.randn(4)
            self.history.append([data_to_pred,])
        else:
            data_to_pred = self.state * data_to_gene
            self.history[-1].append(data_to_pred)

        if self.counter % 10000 == 0:
            print(f"Generator rank{self.rank}: iteration {self.counter} finished.")    #TODO remove
          debug later

        self.counter += 1

        # stop_run should be returned as a bool value
        # data_to_pred should be returned as an 1-D numpy array
        return stop_run, data_to_pred

    def stop_run(self):
        """
        Called before the Generator process terminating when active learning workflow shuts down.
        """
        ##### User Part #####
        self.save_progress()
```

S7 Oracle kernel

Each process in the Oracle kernel is started with an MPI rank and a working directory (`result_dir`). Once the PAL is shutdown by any Training process or Generator process, the `stop_run` method is called by every Oracle process before quitting.

```
def __init__(self, rank, result_dir):
    """
    Initilize the model.

    Args:
        rank (int): current process rank (PID).
        result_dir (str): path to directory to save metadata and results.
    """
    self.rank = rank
    self.result_dir = result_dir

def stop_run(self):
    """
    Called before the Oracle process terminating when active learning workflow shuts down.
    """
    ##### User Part #####
    pass
```

Oracle processes generate ground-truth labels for ML model training by repeatedly calling the `run_calc` method with a 1-D arry (`input_for_orcl`) as input, which is selected by the `prediction_check` function (list_input_to_orcl). The input data (`input_for_orcl`) and ground-truth label (`orcl_calc_res`) are then collected by Controller before broadcasting to Training processes (Figure 1, flow in green).

```
def run_calc(self, input_for_orcl):
    """
    Run Oracle computation.
    Args:
        input_for_orcl (1-D numpy.ndarray): input for oracle computation.
                                            Source: element of input_to_orcl_list from utils.
     prediction_check()

    Returns:
        orcl_calc_res (1-D numpy.ndarray): results generated by Oracle.
                                           Destination: element of datapoints at UserModel.
     add_trainingset().
    """
    orcl_calc_res = None
    ##### User Part #####
    orcl_calc_res = np.random.randn(4,)

    # orcl_calc_res should be returned as an 1-D numpy array
    return orcl_calc_res
```

Utilities

The `prediction_check` function takes as arguments a list of predictions gathered from the Prediction kernel and a list of inputs gathered from the Generator kernel, and selects inputs that are sent to the Oracle kernel for labeling by user-defined criteria (`list_input_to_orcl`, e.g. standard deviation of ensemble model predictions). Data returned as `list_data_to_gene_checked` are scattered to Generator processes for the next AL iteration. Notice that the size of `list_data_to_gene_checked` and the order of 1-D arrays in it should match the corresponding processes in the Generator kernel.

```
def prediction_check(list_data_to_pred, list_data_to_gene):
"""
User defined predictions check function.
Check the predictions from Prediction processes (e.g. STD).

Args:
    list_data_to_pred (list): list of data_to_pred gathered from all generators, sorted by the
     rank of generator.
                              Source: list of data_to_pred from UserGene.generate_new_data()
                              [1-D numpy.ndarray, 1-D numpy.ndarray, ...], size equal to number
      of generators.
    list_data_to_gene (list): list of data_to_gene gathered from all models in prediction
     kernel, sorted by the rank of model.
                              Source: data_to_gene_list from UserModel.predict()
                              [numpy.ndarray, numpy.ndarray, ...], array shape (n_pred, model
     output size), size equal to number of generators.

Returns:
    list_input_to_orcl (list): list of user defined input to oracle to generate ground truth.
                               Destination: list of input_for_orcl at UserOracle.run_calc().
                               [1-D numpy.ndarray, 1-D numpy.ndarray, ...]
    list_data_to_gene_checked (list): list of predictions distributed to generators.
```

```
                              Destination: list of data_to_gene to UserGene.generate_new_data()
    , length must match the number of generators and should be sorted by the rank of generator.
                              [1-D numpy.ndarray, 1-D numpy.ndarray, ...]
"""
list_input_to_orcl = []
list_data_to_gene_checked = []

##### User Part #####
threshold = 0.0  # set the threhold for standard deviation (std)

list_data_to_gene = np.array(list_data_to_gene, dtype=float)
std = np.std(list_data_to_gene, axis=1, ddof=1)  # calculate std of PL predictions
# identify PL input with high prediction std
i_orcl = np.where((std > threshold).any(axis=1))[0]
list_input_to_orcl = [list_data_to_pred[i] for i in i_orcl]

# limit the growth of list_input_to_orcl in this specific example to save memory
i = np.random.randint(0, 2)
list_input_to_orcl = list_input_to_orcl[:i]

pred_list = np.mean(list_data_to_gene, axis=1)  # take the mean of predictions to send to
    generator
pred_list[i_orcl] = 0  # for predictions with high std, send 0 instead to generator
list_data_to_gene_checked = list(pred_list)

return list_input_to_orcl, list_data_to_gene_checked
```

It might be desired to periodically adjust data waiting for labeling with the most up-to-date ML model to save the Oracle resources. This can be achieved by implementing the `adjust_input_for_oracle` function that takes as inputs the list of data for labeling (`to_orcl_buffer`) and corresponding predictions from the most up-to-date ML models in the Training kernel (`pred_list`). The order of inputs in the buffer could be altered and some inputs could be removed according to the predictions. This function is turned on by setting the `dynamic_orcale_list` to `True` in `al_setting`.

```
def adjust_input_for_oracle(to_orcl_buffer, pred_list):
"""
User defined function to adjust data in oracle buffer based on the corresponding predictions in
     pred_list.
Called only when dynamic_orcale_list is True in al_setting.

Args:
    to_orcl_buffer (list): list of input for oracle labeling.
                           Source: list of input_to_orcl to UserOracle.run_calc().
                           [1-D numpy.ndarray, 1-D numpy.ndarray, ...], size equal to number of
     elements in the oracle buffer
    pred_list (list): list of corresponding predictions of to_orcl_buffer from retrained ML.
                      Source: UserModel.predict()
                      [1-D numpy.ndarray, 1-D numpy.ndarray, ...], size equal to number of
    elements in the oracle buffer
Returns:
    to_orcl_buffer (list): list of adjusted input for oracle labeling. (list of input_to_orcl
    to UserOracle.run_calc())
                           Destination: list of input for oracle labeling.
                           [1-D numpy.ndarray, 1-D numpy.ndarray, ...]
"""

##### User Part #####
threshold = 0.0  # set the threhold for standard deviation (std)

std = np.std(np.array(pred_list, dtype=float), axis=0, ddof=1)  # calculation std of
    predictions from retrained ML
# sort the to_orcl_buffer list based on the std
i_orcl_sorted = np.argsort(np.mean(std, axis=1), axis=0)[::-1]
to_orcl_buffer = np.array(to_orcl_buffer, dtype=float)[i_orcl_sorted]
std = std[i_orcl_sorted]
to_orcl_buffer = list(to_orcl_buffer[np.nonzero((std > threshold).any(axis=1))[0]])  # remove
    data with prediction std not exceeding the threshold

return to_orcl_buffer
```
